# Territoriality and variation in home range size through the entire annual range of migratory great spotted cuckoos (*Clamator glandarius*)

**DOI:** 10.1038/s41598-019-41943-2

**Published:** 2019-04-17

**Authors:** Josse Rühmann, Manuel Soler, Tomás Pérez-Contreras, Juan Diego Ibáñez-Álamo

**Affiliations:** 1 Behavioural and Physiological Ecology group, Centre for Ecological and Evolutionary Studies, University of Groningen, 9700 CC Groningen, The Netherlands; 2 Present Address: Department of Zoology, Faculty of Sciences, University of Granada, E-18071 Granada, Spain

**Keywords:** Behavioural ecology, Animal behaviour

## Abstract

Variation in home range size throughout the year and its causes are not well understood yet. Migratory brood parasites offer a unique opportunity to incorporate this spatio-temporal dimension into the study of the factors regulating home range dynamics. Using satellite transmitters, we tracked sixteen migratory great spotted cuckoos (*Clamator glandarius*) of both sexes for up to three years. We constructed home ranges in all major staging areas, from the Spanish breeding areas to the African wintering grounds, analyzed their temporal and geographical variation and investigated their main potential determinants (e.g. food and host availability). We found that home ranges were significantly larger in the breeding area compared to non-breeding areas. Using NDVI as a proxy for food availability, we showed that breeding area home ranges have significantly lower food availability per km^2^ than home ranges elsewhere which could explain why cuckoos use alternative areas with higher food availability before initiating migration. We also found some evidence for sex differences. Additionally, we found no indications of territoriality in this species, providing novel information into the current debate on brood parasite territoriality. Overall, food availability seems to be an important factor regulating home range dynamics and influencing migratory patterns throughout the year in great spotted cuckoos.

## Introduction

Space use by animals is a key factor in animal ecology and one of its core concepts is the home range^1,2^. A home range is defined as ‘that area traversed by the individual in its normal activities of food gathering, mating and caring for young’^1^, indicating the close relationship between animals and their environment. In fact, a home range can also be defined as the smallest area that contains all the resources an animal requires^3^. Various biological aspects such as behavior, physiology and population density can be linked to home range size, making it an important ecological variable in the study of animals^4^ and crucial for conservation^5,6^.

Several internal factors such as body weight and sex^3^, as well as external factors such as resource availability^2,7^ have been found to influence home range size. In birds, a common model system for the study of movement ecology and home range research, the availability and distribution of food is likely the primary external factor affecting home range size^7,8^. Interestingly, resource availability and animal’s resource requirements can vary throughout the year, often leading to a seasonal variation in home range size^7^. However, studies on the seasonal variation of home range size are still scarce and have shown conflicting results with some of them showing larger home ranges during the breeding season^9,10^ while others finding the opposite pattern^11,12^. In this context, migratory species are particularly interesting because they experience important geographic variation that might affect their home ranges in different ways. Considering that the majority of home range investigations are short-term studies (1–2 months), the entire annual perspective is especially relevant for migratory species^13^ as they will help us to better understand the factors that determine the variation in home ranges. This knowledge of (risk) factors throughout the annual cycle is vital to interpret the population dynamics of vulnerable species and to establish successful conservation efforts.

Migratory brood parasites offer an excellent opportunity to investigate home range size variation throughout the annual cycle and its causative factors. Brood parasites are not burdened by having to feed their offspring given that they lay their eggs in the nests of their host species^14^, and therefore changes in food demands between wintering and breeding areas are unlikely to cause home range size variation in these species. In addition, the only study exploring annual variation in home range size in a threatened brood parasite, the common cuckoo (*Cuculus canorus*), suggests a difference in home range size between their breeding and wintering areas^13^, indicating the importance of alternative factors in explaining this variation. Some studies have suggested that these differences are caused by the need to incorporate areas where their host’s nests are located^13,15^. Since these areas do not necessarily overlap with feeding areas, this could lead to larger home ranges during the breeding season^16^ and explain why brood parasites usually have larger home ranges than same-size non-parasitic species^15,17^. However, following the general trend in home range studies, brood parasites have been almost exclusively studied during the breeding season, stressing the urgent need of a new spatio-temporal dimension to fully comprehend the coevolutionary relationships between them and their hosts^18^.

Territoriality, the defense of a home range from conspecifics to secure the exclusive access to resources, is directly related to home range size^19^. Territoriality is generally expected to occur in brood parasites given that multiparasitism is costly^20^. Nevertheless, territoriality in brood parasites has been shown to differ between species and populations. Pairs of Horsfield’s bronze cuckoos (*Chrysococcyx basalis*) were shown to occupy exclusive breeding areas^21^. For brown-headed cowbirds (*Molothrus ater*) indications of territorial defense in females were found in some^22^ but not in other studies^15^. Extensive overlap of female home ranges during the breeding season (suggesting an absence of territoriality) was shown in other brood parasitic species, including shiny cowbirds (*Molothrus bonariensis*)^23^ and common cuckoos^16,17^. Part of the variation in the occurrence of territoriality in brood parasites could result from different territorial strategies^24^. Parasites could defend a large area and all nests inside with only some overlap at the boundaries (classical territory) or they could defend several smaller patches of nests consecutively, for only the time they can be parasitized (dynamic territory). However, knowledge on brood parasite territoriality relies almost exclusively on studies using females^24^, highlighting the need to also investigate males, as well as possible sex differences. Sex is an important internal factor determining home range size, with females usually having smaller home ranges than males during the breeding period^8^. In fact, home range size can be regulated by different factors for each sex: energy requirement for females and mating opportunities for males^7^, which could also affect territoriality differently.

The main aim of this study is to investigate sex differences in annual variation in home range size of brood parasites and its relationship with food availability and host nest availability. We used the great spotted cuckoo (*Clamator glandarius*) as our model species because it is a long-distance migratory species^25,26^ that has been intensively studied in their European breeding sites^27^ offering a good combination of previously known information (i.e. coevolutionary relationship with its hosts^28,29^ and large geographic variation^30,31^). We predict that (female) home range size is going to be larger in the breeding area than in the wintering grounds given the potential role that host nest availability seems to play in the former and the fact that breeding and feeding areas do not fully overlap^13,15^. Alternatively, food availability could be the major driver of home range size in all geographical regions^7,8^, resulting in a negative correlation between size and food availability. It is very difficult to make predictions regarding sex differences due to the lack of knowledge on movement ecology for male and female brood parasites in general (see above). On the one hand, we might expect no sex differences in home range size as neither sex delivers parental care behaviors. On the other hand, food availability might still be a larger determinant of female home range size due to the energy requirements of laying a large amount of eggs in parasitic females. Another important objective of our investigation was related to territoriality. A previous genetic study found strongly overlapping female laying areas suggesting no territoriality in female great spotted cuckoos^24^, so we predict the absence of territoriality in this species, although it is possible that males can follow a different strategy (see above). We hope that our study can provide new insights into our understanding of the variability of home range size and help integrate a broader spatio-temporal perspective into the study of brood parasitism.

## Results

Using Argos satellite trackers, we obtained data on the movements of 15 cuckoos (9 males, 6 females). Fourteen birds were tracked between 12 days and 10 months (median = 75 days) and another provided information for three years. We calculated a total of 53 home ranges that were distributed in four different general areas: the breeding area (n = 20 home ranges based on information provided by 15 cuckoos), non-breeding areas in Spain (n = 11, 6 cuckoos), stop-over area in Morocco (n = 4, 4 cuckoos) and the wintering area in the Sahel region of Western Africa (n = 18, 3 cuckoos). Additional information on the migratory movements and tracking period of these individuals as well as details on data preparation is available elsewhere^26^.

### Home ranges, geography and sex

**Figure 1:**
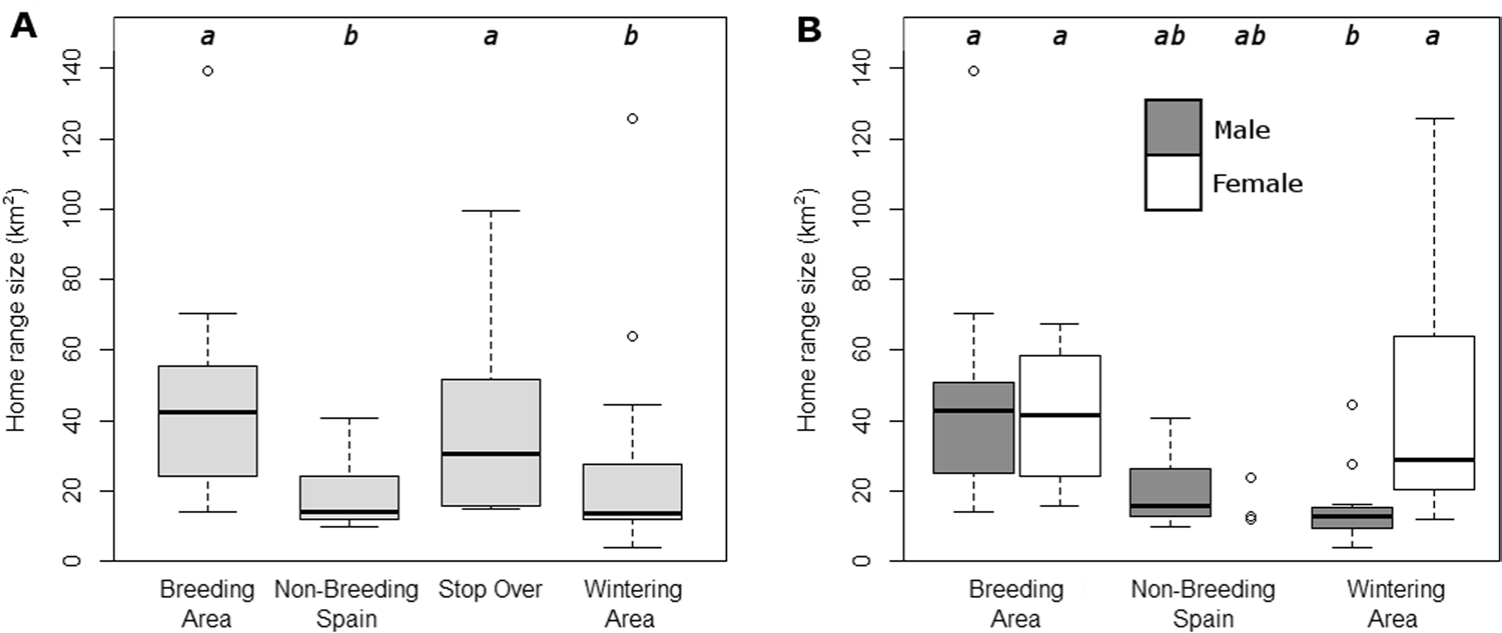
Home range size difference between region and sex. Home range size calculated using 95% kernel density estimation. (**A**) Home range size differences between regions. The number of home ranges (n^h^) used and its corresponding number of cuckoos (n^c^) were the following (n^h^/n^c^): Breeding = 20/15, Non-breeding Spain = 11/6, Stop Over = 4/4, Wintering = 18/3. (**B**) Sex difference in home range size (n^h^/n^c^): Breeding: Male = 13/9, Female = 7/6; Non-breeding Spain: Male = 8/3; Female = 3/3; Wintering Area: Male = 12/2; Female = 6/1. Columns showing different letters indicate significant differences (*p* < 0.05) between them according to Tukey posthoc tests. Since there were only three female home ranges in non-breeding Spain no boxplot was drawn but the individual data points are shown. The stop-over area was not included due to the small sample size.

The median size of all home ranges (95% KDE, *n* = 53) was 25.1 km^2^ (range: 3.9–139.3 km^2^; Table 1). Median core range (50% KDE) size was 3.5 km^2^ (range: 0.7–26.6 km^2^), and individual core ranges were on average 6.4 times smaller as the full home range. There was a significant difference in home range size between regions (*F* = 5.82, *df* = 3, *p* = 0.002; Fig. 1A). Post-hoc analysis revealed that home ranges in the breeding area were significantly larger than home ranges in non-breeding Spain (*p* = 0.018) and the wintering area (*p* = 0.005), but not than those in the stop-over region (*p* = 0.99; Table 1 and Fig. 1A). A similar outcome to the KDE 95% comparison was found for the core ranges (*F* = 5.573, *df* = 3, *p* = 0.002).

**Table 1:** Home range size calculated using 95%, 50% Kernel Density Estimation (KDE), and Minimum Convex Polygons (MCP) in each area for males (M), females (F) or both combined (Com).

		KDE				n^h^	n^c^	MCP			
		95%		50%							
		Median	Range (km²)	Median	Range (km²)			Median	Range (km²)	n^h^	n^c^
Breeding Area	Com	42.3	14.1–139.3	5.6	2.5–26.6	20	15	58.6	1.9–219.5	22	15
	M	41.6	14.1–139.3	5.5	2.5–26.6	13	9	58.6	8.0–219.5	14	9
	F	42.9	15.8–67.4	6.2	2.6–10.4	7	6	53.5	1.9–100.0	8	6
Non-Breeding Spain	Com	14.0	9.9–40.5	2.5	1.4–5.4	11	6	18.0	5.6–45.4	14	8
	M	15.7	9.9–40.5	2.7	1.4–5.4	8	3	18.4	7.0–45.4	11	5
	F	12.1	12.0–23.6	2.2	2.0–3.3	3	3	8.3	5.6–23.1	3	3
Stop-Over	Com	40.9	15.6–99.4	6.5	2.9–20.9	4	4	17.5	10.0–122.9	5	5
	M	23.0	15.6–30.6	4.1	2.9–5.3	2	2	13.8	10.0–17.5	2	2
	F	75.5	51.5–99.4	14.3	7.7–20.9	2	2	37.4	11.5–122.9	3	3
Wintering Area	Com	13.6	3.9–125.5	2.3	0.7–17.4	18	3	9.5	1.2–162.6	34	3
	M	12.8	3.9–44.5	2.2	0.7–9.1	12	2	7.1	1.2–35.2	26	2
	F	28.9	11.7–125.5	5.0	2.2–17.4	6	1	17.8	3.4–162.6	8	1
Combined	Com	25.1	3.9–139.3	3.5	0.7–26.6	53	15	16.3	1.2–219.5	75	15
	M	18.1	3.9–139.3	2.9	0.7–26.6	35	9	12.2	1.2–219.5	53	9
	F	31.5	11.7–125.5	5.1	2.0–20.9	18	6	22.5	1.9–162.6	22	6

The column n^h^ indicates home range sample sizes, while n^c^ refers to cuckoo sample sizes.

Males and females had similar home range sizes overall (*F* = 2.83, *df* = 1, *p* = 0.10), but sex did show a significant interaction with region (excluding stop-over sites due to insufficient sample size; *F* = 4.005, *df* = 2, *p* = 0.025; Fig. 1B). Post-hoc tests showed that there was a sex difference in the wintering area (*p* = 0.025), but not in the breeding area (*p* = 0.99) or non-breeding areas in Spain (*p* = 0.99; Table 1 and Fig. 1B). In the wintering area females home ranges were significantly larger than those of males.

### Home ranges and food availability

**Figure 2:**
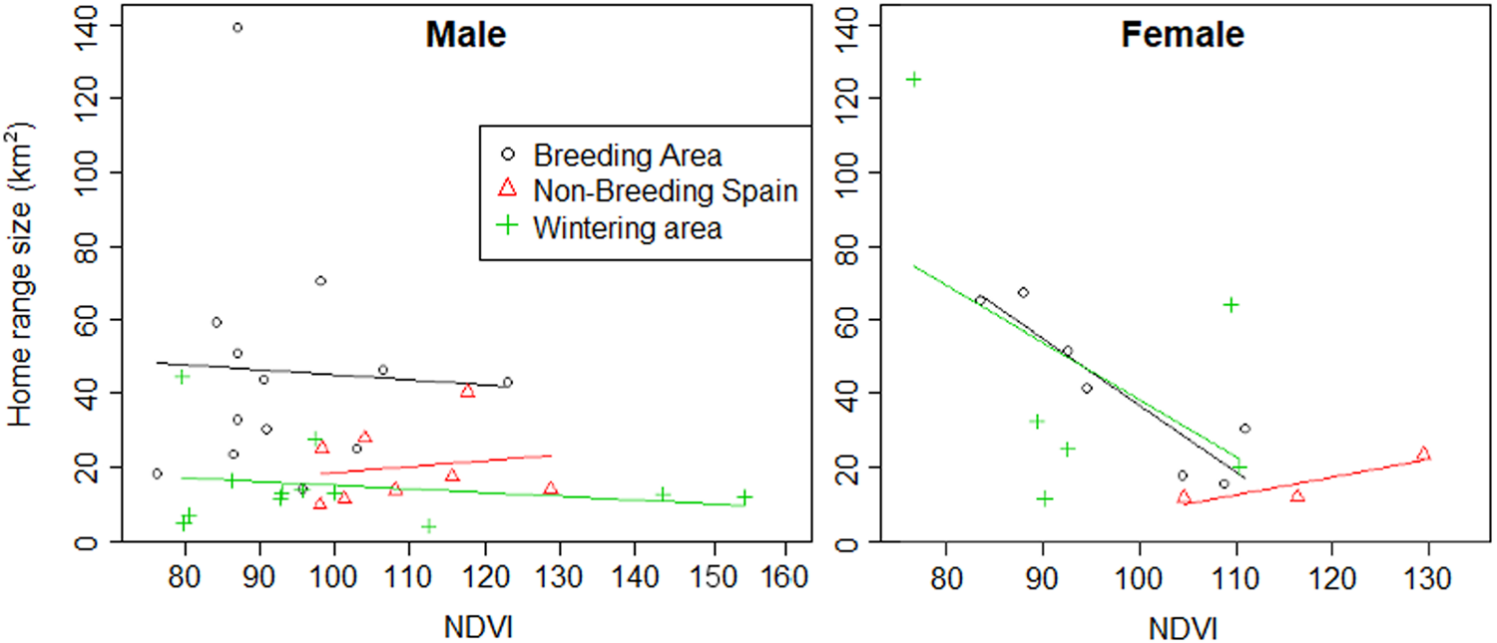
Relationship between KDE 95% home range size and NDVI by region and sex. The home ranges used in this figure correspond to the following number of cuckoos: Breeding: Male = 9, Female = 6; Non-breeding Spain: Male = 3; Female = 3; Wintering Area: Male = 2; Female = 1.

**Figure 3:**
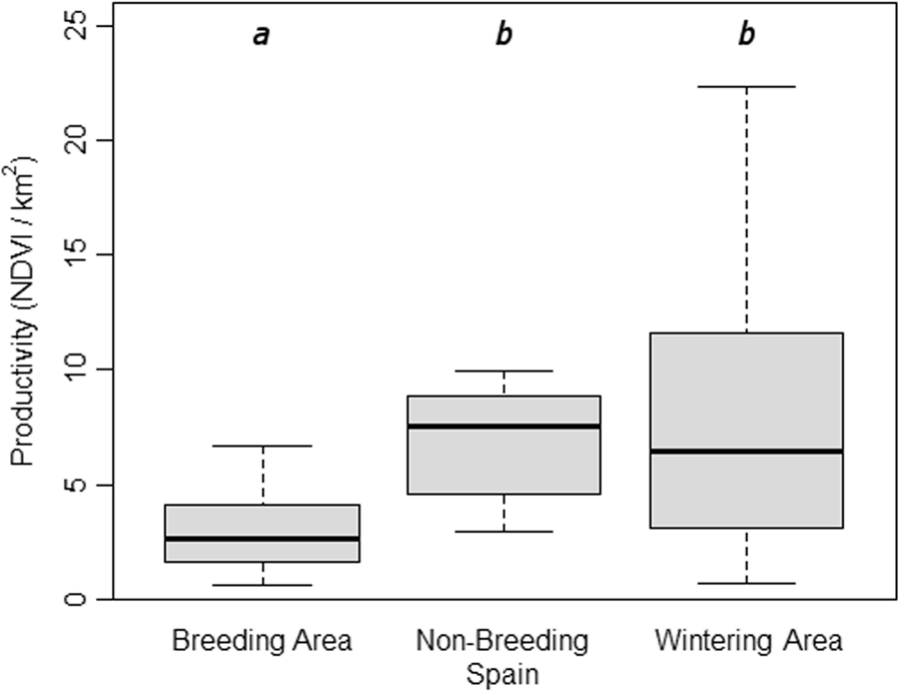
Differences in home range NDVI/km^2^ by region. Columns with different letters indicate significant differences (*p* < 0.05) between them according to Tukey posthoc tests. The number of home ranges (n^h^) used and its corresponding number of cuckoos (n^c^) were the following (n^h^/n^c^): Breeding = 20/15, Non-breeding Spain = 11/6, Wintering = 18/3.

We used the Normalized Difference Vegetation Index (NDVI), a graphical indication of the presence of green vegetation, as a proxy for food availability^32^. We found no significant relationship between the size and the average NDVI value of individual home ranges either overall (*t* = −0.95, df = 51, *p* = 0.34), or in any separate region (Breeding area: *t* = −0.91, *p* = 0.37; Non-breeding Spain: *t* = 0.53, *p* = 0.60; Wintering area: *t* = −0.24, *p* = 0.81; Stop-over: *t* = 0.33, *p* = 0.74). However, this effect differed between sexes (Fig. 2). Only for females in the breeding area there was a significant relationship between home range size and home range NDVI (*t* = −2.477, *p* = 0.033). Home range NDVI/km^2^ was significantly different between regions (excluding stop-over: *F* = 7.226, *df* = 2, *p* = 0.002; Fig. 3). Post-hoc comparisons showed that breeding area home ranges have significantly lower NDVI/km^2^ than those in non-breeding Spain (*p* = 0.030), and the wintering area (*p* = 0.002). There was no significant relationship between breeding home range size and the body mass of the birds (*t* = −0.68, *df* = 12, *p* = 0.51).

Most cuckoos switched between home ranges within a region, some of them multiple times. Breeding home range NDVI was not predictive of whether cuckoos left or stayed (*t* = 1.63, *df* = 14,8, *p* = 0.12). However, birds that left the breeding area for other areas in Spain increased their home range NDVI by an average NDVI value of 20.9 ± 6.3 (*n* = 10). Four cuckoos that relocated to a new home range within the breeding area increased their home range NDVI by 11.0 ± 1.6. Subsequent switches did not have the same effect (NDVI −2.1 ± 6.4, *n* = 5). In total, home range switching within Spain resulted in a significant increase of home range NDVI by 12.8 (t = 3.651, p = 0.002), and there were only 3 switches to a home range with a substantially lower NDVI value (<5). This was not the case in the wintering area, where overall, switches resulted in a non-significant decrease of home range NDVI by 3.6 ± 23.2 (*n* = 25, *t* = −0.79, *p* = 0.44). Only 5 switches in Africa resulted in a clear increase in home range NDVI, while 11 resulted in a clear decrease (*X*^2^ = 2.30, *p* = 0.13).

**Figure 4:**
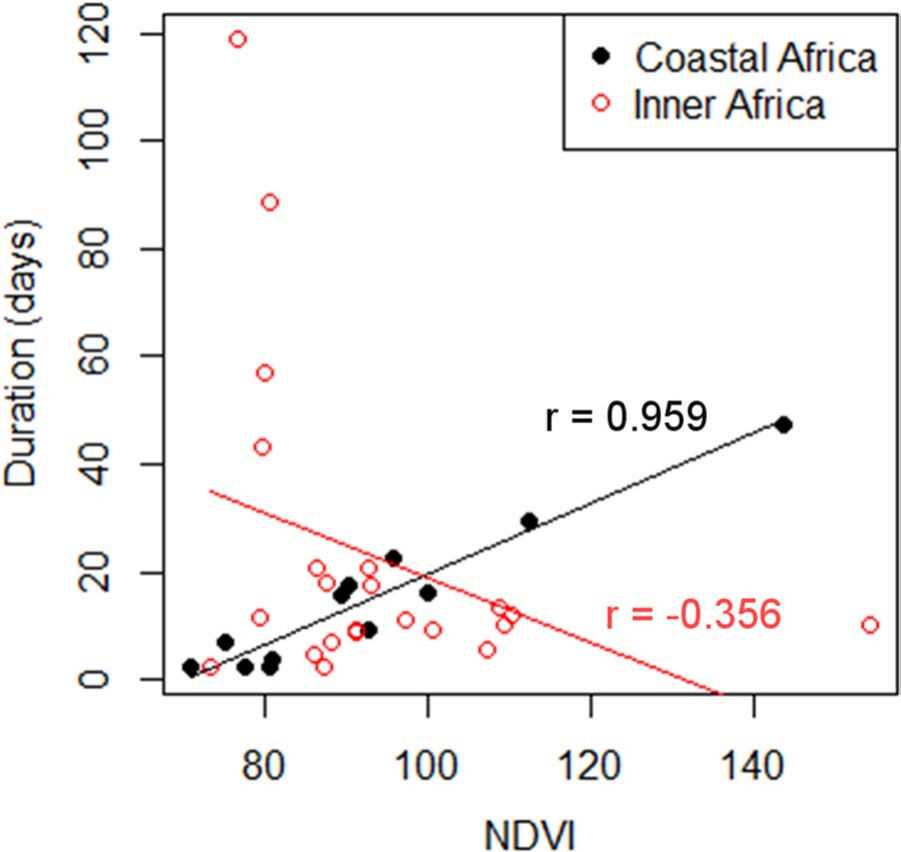
Relationship between NDVI and duration of stay in the wintering area. Each dot represents a home range. The information presented in this figure corresponds to that provided by three cuckoos both in Coastal and Inner Africa.

There was a strong positive relationship between home range NDVI values and duration of stay in the coastal region of the wintering grounds (*r* = 0.96; *t* = 4.790, *p* = 0.0007), but not in inland regions (*r* = −0.35; *t* = −0.98, *p* = 0.34; Fig. 4). In inner Africa, this correlation was absent even when removing the final wintering home ranges of the cuckoos (where they spent the longest period of time), which all had similar relatively low NDVI values of around 80 (*r* = 0.06, *t* = 0.95, *p* = 0.36).

### Home ranges and host availability

**Figure 5:**
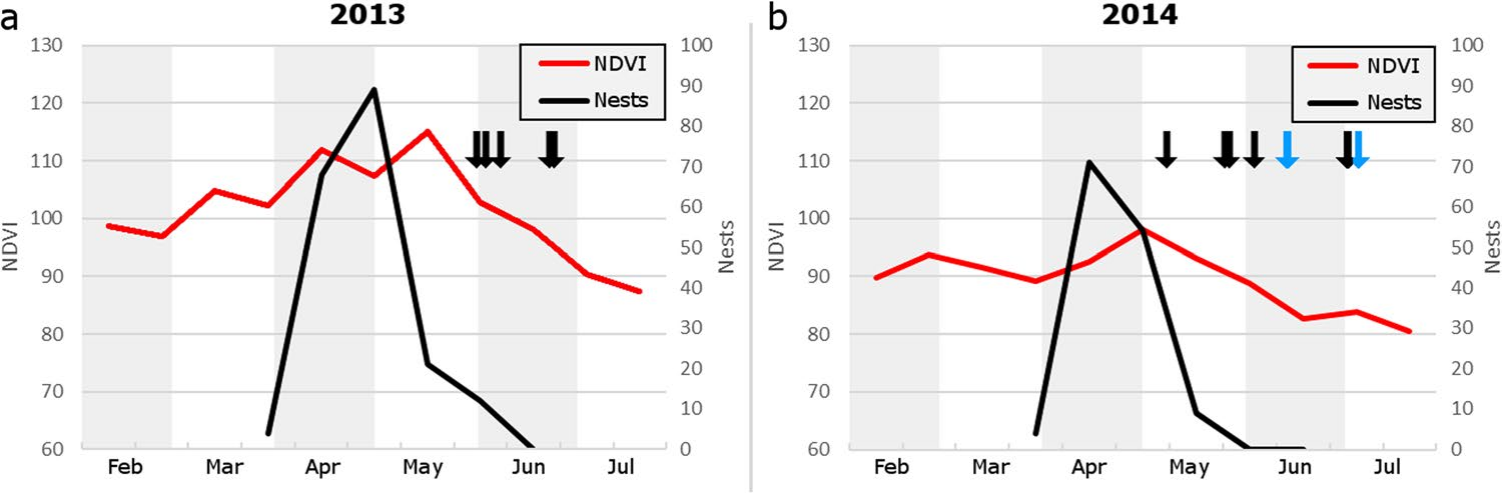
Changes in NDVI and magpie nests in the breeding area for 2013 (**a**) and 2014 (**b**). Both explanatory variables were calculated over the same biweekly periods. Arrows indicate when cuckoos left the breeding area. Black arrows = cuckoos that left for other areas in Spain, blue arrow = cuckoos that left for post-breeding migration.

Nests of the cuckoo’s main hosts in the breeding area, Eurasian magpies (*Pica pica*), were surveyed throughout the breeding season. Great spotted cuckoo home range sizes in the breeding area were, unexpectedly, much larger than those found in previous studies^33^. This meant that all home ranges extend beyond the magpie nesting-survey area, which precluded testing for any individual effects with regards to the number of nests per home range. Therefore, only a population level analysis was performed on the relationship between cuckoo and magpie laying strategies. Observations in the study area during the breeding period showed that the magpie laying period starts in early April and lasts until mid-May/early June, with the peak occurring during the second half of April (Fig. 5). The great spotted cuckoo laying period closely matches this. The highest NDVI values in the breeding area occurred shortly after the magpies peak laying period and declined afterwards. Overall NDVI values of the breeding area were significantly lower in 2014 compared to 2013 (*t* = 6.944, *df* = 10, *p* < 0.0001).

### Home range predictor model

To see which parameter best explains home range size (KDE 95%), we run generalized linear mixed-effect models (GLMM). According to our selection procedure (Table 2), the model with the lowest AICc included region, sex and their interaction, in which region (*X*^2^ = 9.929, *df = *2, *p = *0.007) and the interaction term (*X*^2^ = 9.341, *df* = 2, *p* = 0.009) were significant, but not sex alone (*X*^2^ = 0.60, *df* = 1, *p* = 0.44). The model that only included region is equally parsimonious with a ΔAICc < 2 (Table 2)^34^. This mirrors the results described in the previous paragraphs, with region being the most important parameter. Both nuisance factors were highly significant (sample size: *X*^2^ = 58.407, *df* = 1, *p < *0.0001; mean quality of fixes: *X*^2^ = 11.268, *df* = 1, *p* = 0.0008), indicating that these factors play an important role in determining home range size using 95% KDE and that it is justified to account for them in our models.

**Table 2:** Model selection results for the Generalized Linear Mixed-Effects Models explaining (95% KDE) home range sizes and including region, sex and home range NDVI as explanatory variables.

Model Parameters	AICc	Δ AICc	k
region + sex + region*sex	42.3	—	5
region	44.0	1.7	3
region + sex + NDVI + region*sex	45.6	3.3	6
region + sex	46.2	3.9	4
region + NDVI	46.3	4.0	4
region + NDVI + region*NDVI	47.2	4.9	5
*Null model*	49.7	7.4	2

The models also include sample size and mean quality of fixes as nuisance variables, as well as bird identity as a random factor.

### Absence of breeding territoriality

**Figure 6:**
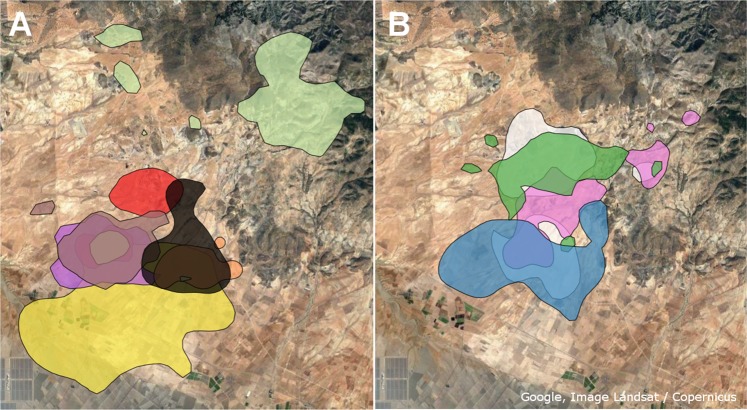
Core home ranges (50% KDE) of males (**A**) and females (**B**) in the breeding area during the breeding period (Mar–Jul) in 2014. Colors correspond to different individuals. The satellite imagery was obtained from Google Earth 7.1 (Google Inc., CA, USA).

Core home ranges (50% KDE) in the breeding area showed a great amount of overlap and gave little indication for territoriality (Fig. 6). In 2014, among 11 individuals there was on average 21.1% overlap in core home ranges between any pair of cuckoos of the breeding area. On average each cuckoo’s core home range overlapped with that of 7.7 others, shared more than 30% of their core home ranges with 2.8 cuckoos, and over 50% with 1.4 cuckoos. Separating sexes showed stronger overlap in females (27.9%, *n* = 4; males: 18.8%, *n* = 7). In 2013, overlap was much less strong (7.7%) but this information is only based on 4 cuckoos. Outside the breeding area there was only one incidence of overlap of home ranges: between two males in the region near Albacete. There was no overlap between home ranges of different individuals in the stop-over region or the wintering grounds.

## Discussion

We found that great spotted cuckoo home ranges in the breeding area were larger than those in the main non-breeding areas (Fig. 1A), matching our prediction. This pattern was observed for both male and female cuckoos (Fig. 1B). Modeling the influence of several variables on home range size showed that region is the best predictor of home range size (Table 2). Common cuckoos show a similar change in home range size between breeding and wintering areas, although the size difference was much greater for that species^13^. In the breeding area, great spotted cuckoos have smaller home ranges (mean ± sd: 44.4 ± 28.5 km^2^; n = 20) than common cuckoos (136.1 ± 70.0 km^2^; n = 11) (*t* = 4.157, *p* = 0.001) while the opposite is found outside the breeding area (25.9 ± 25.9 km^2^ versus 12.4 ± 8.2 km^2^ for great spotted (n = 34) and common cuckoos (n = 63) respectively; *t* = −2.958, *p* = 0.005). These contrasting results in home range size between regions were also found for other non-parasitic species. For instance, in the breeding area, some studies have found smaller home ranges^11,12,35^ while others have shown larger home ranges^9,10^. Overall changes in food availability and food requirements, usually related to chick demands, have been commonly used to explain those differences although other factors such as intraspecific competition or population size seem to play an important role as well^10^.

Our results showing different home range sizes in each region are in accordance with the hypothesis that having to incorporate an additional resource (host nests) into the home range during the breeding period increases overall home range size, possibly because of a mismatch between optimal foraging and host breeding areas^13^. Unfortunately, we could not test this hypothesis directly, although it seems to be supported by the finding that the productivity/km^2^ of the breeding home ranges is significantly lower than that of the non-breeding home ranges in both Spain and Africa. Other species such as the common cuckoo^17,36^ and brown-headed cowbirds^15,37^ solve this problem by having enlarged home ranges that encompass distinct breeding and feeding areas. There was no indication that this was the case for great spotted cuckoos, as they can feed in the same areas where they parasitize their magpie hosts. Interestingly, our data suggests that cuckoos might be using a temporal strategy and could explain the existence of non-breeding areas in Spain (sometimes further north) that are used between breeding and migration^26^. Many individuals leave the breeding area for non-breeding areas in Spain with significantly higher productivity (NDVI values) (Fig. 3), which suggests the use of suboptimal foraging areas during the laying period. Subsequently, as soon as the laying period is finished, optimal foraging areas become the primary determinant of home range selection which led them to these non-breeding areas in Spain. Alternatively, home ranges in the breeding area could be larger due to lower habitat quality^13^, independently of host nest availability.

Interestingly, the regional differences in home range size varied with sex (Table 2). In the breeding area, males and females have similar-sized home ranges, a finding in line with the non-significant results found in the majority of avian studies^8^. In the wintering grounds however, female home ranges were larger (Fig. 1B). The small sample size for non-breeding areas in Spain and stop-overs prevent us from making conclusions for those regions. However, the results for the breeding and wintering areas suggest that the requirements for each sex vary with region. Similar findings in white-crowned sparrows (*Zonotrichia leucophrys*)^38^ and houbara bustards (*Chlamydotis undulata*)^39^ were thought to be related to social organization and mating systems. Food availability could also be important in this context as females might need more resources in the wintering areas to replenish their fat reserves and prepare for the next breeding season, which, due to the high fecundity of cuckoos^40^, is more energy demanding than for males. However, caution should be taken with some of these results since all female data from the wintering area came from one individual. Another interesting sex difference is that we found a significant negative relationship between productivity and home range size in the breeding area only for females (Fig. 2). This implies that food availability is more important for females than for males in determining home range size during the laying period, which fits with our prediction.

Our data also gives some insights into the strategies followed by different individuals after the egg laying period. While the majority of individuals leave the breeding area for other regions, some cuckoos stay in the breeding area until post-breeding migration^26^. It has been recently suggested that those individuals that move to alternative areas in Spain could do so to parasitize additional host populations, to prospect for future breeding locations or to refuel before the post-breeding migration^26^. Our results support the latter hypothesis as Spanish non-breeding home ranges have a higher productivity than those in the breeding area. Additionally, home ranges in these new areas were significantly smaller than those in the breeding area, contrary to what would have been expected if they were parasitizing hosts in both areas. Our results also suggest that cuckoos use highly productive coastal areas in the wintering grounds in order to replenish their energy reserves after their trans-Saharan trip. The strong relationship between the duration of stay in these coastal home ranges and food availability (productivity; Fig. 4) indicates an important role of this factor in this particular post-migratory period. Similar findings of a relationship between itineracy in the wintering regions and local NDVI were found for various species^41–43^. The absence of this relationship in inland Africa indicates that other factors besides food availability, such as predator avoidance, may play a more important role in determining habitat quality for cuckoos in that region^8^.

Regarding territoriality, we found little indication for cuckoo territoriality in the breeding area as there was considerable overlap between any pair of core home ranges (females: 27.9%, males: 18.8%; Fig. 6). While some cuckoos had largely non-overlapping home ranges, not all cuckoos in the study area were tagged, suggesting that even though the percentages might seem low, they could be underestimating the real overlap. This could also be occurring in more peripheral areas since tagged cuckoos were mostly caught in the center of the breeding area. Alternatively, these areas could represent less suitable habitats that are mainly occupied by cuckoos that are not able to compete with others for the more preferred areas^44^. Our results contrast with those of a previous observational study based on unmarked individuals and egg morphology supporting that great spotted cuckoos have territories^33^. However, they match with the findings of another study using molecular methods indicating that this species does not have exclusive laying territories and that multiparasitism occurs regularly in the cuckoo-magpie system^24^. In fact, our findings support the hypothesis that there is no territoriality in this species^24^.

Economic indefensibility of home ranges could lead to a lack of territoriality^45^ and could explain it in brood parasites. Large home ranges increase the costs of defense^16^ and it has been suggested that this is the case in the great spotted cuckoo^24^ given that they select the nests to parasitize based on the host quality^46^. On the other hand, the relatively low cost of multiparasitism for great spotted cuckoo chicks’ survival^47^ and the benefits that it provides during the fledgling stage^48,49^ could prevent the evolution of territoriality in this species. This cost could determine whether brood parasites might evolve territoriality and could explain why some evictor species like the Horsfield’s bronze cuckoo defend territories^21^. Nest availability could also play a role in this context. A large number of nests in the study area remained unparasitized, therefore nest availability may be sufficient to accommodate the current number of cuckoos in this population without the need for territoriality. This might imply that territoriality could be more plastic in this species and could vary between years and even breeding areas, as it happens with the level of multiparasitism^24,47,50^. Our data however shows that home ranges overlap in both study years and therefore not supporting the temporal variation in territoriality.

In conclusion, we found that the size of great spotted cuckoo home ranges varies among regions, being larger in the breeding area than in the main non-breeding areas. Our results show sex differences only in the wintering areas, with larger home ranges for females which could indicate different feeding requirements or strategies in Africa. Additionally, our data suggests that food availability (NDVI) does not seem to determine male’s home range size in this species while it seems to be important for female’s home ranges in the breeding and wintering areas, probably indicating higher nutritional and energetic needs for this sex. Furthermore, food availability seems to influence the migratory pattern since cuckoos leave the breeding area for higher productive (NDVI/km^2^) non-breeding areas (in Spain) and they spend more time in the most productive coastal areas in Africa, probably to refuel their energy reserves after the long trans-Saharan migration. Finally, our results provide little support for the existence of territoriality in this cuckoo species and present new information on the spatial behavior of this brood parasite outside the more intensively and traditionally studied breeding period.

## Materials and Methods

### Study area and species

We captured 16 great spotted cuckoos (10 males, 6 females) from a population in the Hoya de Guadix in southern Spain (37°16′ N, 3°00′ W) using mist-nets and playback of conspecific calls during April and May of 2013 (n = 5) and 2014 (n = 11)^26^. The Hoya de Guadix is a high-altitude plateau (approx. 1,000 m a.s.l.) with extensive non-cultivated areas, cereal crops (especially barley), some areas with dispersed holm-oak trees (*Quercus rotundifolia*) and groves of almond trees (*Prunus dulcis*) and pines (*Pinus halepensis* and *P. pinaster*)^48^. Magpies are the main hosts of great spotted cuckoos in this area^50^. Cuckoos were ringed and a drop of blood was taken from the brachial vein and stored in absolute ethanol for molecular sexing^51^. The total duration of capture was less than 15 minutes.

### Transmitters & data preparation

Solar charged Argos Platform Terminal Transmitters (PTTs; Microwave Telemetry Inc.) were attached to the cuckoos as a backpack using a body harness made of Teflon^52^. PTTs weighed 5 g, corresponding to an average of 3.1% (2.8–3.4%) of the cuckoos’ body weight^53^. PTTs were set to record in a 12h-on 48h-off transmission cycle in 2013, and an 8h-on 15h-off cycle in 2014. Only high quality Argos location fixes (Argos quality 3-1, accuracy <1.5 km) were used for analysis. The data was filtered to only include fixes with a unique combination of date, time and location using R (version 3.2.4)^54^. Due to an issue likely caused by different time zones or changes from winter to summer time, many fixes were duplicated only with an exact 1 h or 2 h difference. Since the coordinates received were highly precise, the likelihood that these replicate fixes were accurate representations of the location of the bird is unlikely and all but the earliest fix were removed (Table S1).

To prevent spatial autocorrelation and ensure independence of data, the number of fixes can be reduced^17,55^. However, removing too many fixes can lead to a loss of valuable data when these fixes represent a hotspot in the home range, a problem which has been argued to result in a greater bias than autocorrelation^56,57^. Since the collected data was relatively clumped in time due to the periodic nature of satellite passing, a certain measure of spatial autocorrelation was deemed appropriate. Fixes that were recorded within 5 min from each other were filtered, with only the earliest fix being included. This limit was chosen to ensure that the most serious spatial autocorrelation is eliminated, while not leading to a large loss of valuable information (Table S1).

### Home range analysis

Home ranges were determined for each location where a cuckoo was present for at least two days (representing a minimum of two transmission cycles). Fixes recorded during brief trips away from the home range (>15 km) were excluded from home range calculation, since these areas of incidental visits were not deemed to be part of the actual home range^57^ (Table S1). All location fixes within a 15-km radius were considered to be part of the same home range unless, based on visual inspection and the exact timing of fixes, clear and distinct clusters in space and time could be distinguished, in which case each cluster was taken to reflect a separate home range^13^. In these cases, creating a single home range would exclude valuable temporal information and therefore the determination of multiple home ranges within one region was deemed most appropriate^13,58^. One bird did not produce enough data points to perform any home range analysis. In total 82 home ranges were identified for the remaining 15 cuckoos (Table S2, mean quality class per home range = 1.9). Home ranges were located in four general regions: the breeding area in the Hoya de Guadix, other regions in Spain (non-breeding Spain), the stop-over region during migration (in Morocco) and the wintering area in the Sahel region of Western Africa (which can be further subdivided in a coastal and an inland (>150 km from the coast) region; for detailed information see^26^). The stop-over region only includes post-breeding information as all stop-overs during the pre-breeding period belong to a single individual. The duration of stay of the cuckoos in each home range was determined using the minimum duration the bird was in the area^52^. Due to individual PTT settings (especially in 2013) or an inability to connect with a satellite, PTTs did not record a location on some days. Consequently, the exact dates of departure and arrival from each location were not always available. Home range shapes and sizes were estimated using the ‘adehabitatHR’ package in R^59^.

Kernel Density Estimation (KDE) was the main technique used to determine home ranges (Fig. S1). To represent the full home range, a 95% confidence region was used, which reflects the area associated with a 95% probability of finding the animal. A 50% confidence region was also calculated, reflecting the core area of the home range. Since KDE needs a minimum of 30 locations to accurately calculate a home range^60^, 28 home ranges that did not matched this criteria were excluded from KDE analysis. This filtering left 53 home ranges for further analysis (Table S2, median: 64, range: 32–314 fixes per home range). Using *h*^*ref*^ as the smoothing parameter^61^ resulted in considerable oversmoothing, incorporating large areas with no observed points into the home range. Using *h*^*LSCV*^, home ranges showed a certain amount of undersmoothing, resulting in multiple isolated points being part of the home range (Fig. S1). Considering this technique was successfully used in previous studies (e.g.^13,58^), and these home ranges were for an avian species, this disconnectedness was not seen as problematic. A fixed bivariate normal kernel was used and the grid size was set at 200 m. Additionally, 95% Minimum Convex Polygons (MCP) were calculated to be able to compare certain measures between the largest possible subset of home ranges. Only 3 home ranges with less than 5 fixes were excluded from this analysis. Since KDE 95% is assumed to result in the best approximation of the cuckoo’s home range, all further mentioned sizes are calculated using this method unless otherwise specified.

### Food availability, breeding success and territoriality

Normalized Difference Vegetation Index (NDVI), a graphical indication of the presence of green vegetation, was used as a proxy for food availability^32^. NDVI reflects primary productivity and has been shown to correlate with the distribution and behavior of numerous species^32^. NDVI data, aggregated over 2-week periods, was collected from the IRI/LDEO Climate Data Library^62^ for all areas where home ranges were located (cell size = 250 m). Using QGIS^63^, average NDVI was calculated for each home range (both KDE 95% and MCP) based on the dates the bird was in the area. The average NDVI/km^2^ per home range was calculated by dividing the NDVI score by the size of each home range and compared across regions. To see whether home range switches resulted in a change of home range NDVI, the difference in NDVI between both home ranges at the time of the switch was calculated. We used MCP home ranges for this analysis because they offer a similar relationship with NDVI as KDE home ranges (see below) and offer a larger sample size. Finally, we created an MCP of all breeding area fixes to compare overall breeding area NDVI between years.

We looked for magpie nests in the breeding study area (Hoya de Guadix) during the breeding seasons of 2013 and 2014. We surveyed all found magpie nests every other day to record the start and end dates of the laying period as well as the number of magpie and cuckoo eggs. The number of parasitized magpie nests in the breeding area was similar between years (2013: 87; 2014: 85). However, since there were less magpie nests overall in 2014 (181 vs. 117), the rate of parasitism was much higher that year (2013: 48.1%, 2014: 72.6%; *X*^2^ = 16.605, *p* < 0.0001). The number of cuckoo eggs was higher in 2014 too (169 vs. 213) resulting in a higher average number of cuckoo eggs per nest (1.9 vs. 2.5). Nevertheless, the percentage of successfully fledging cuckoos was similar (2013: 31.5%, 2014: 36.3%; *X*^2^ = 0.63, *p* = 0.43). To be able to make comparisons with the biweekly NDVI data, the number of laying magpies (and consequently potential laying opportunities for cuckoos) in the area was calculated for the equivalent two-week periods.

Territoriality was assessed by computing the amount of overlap between all pairs of core home ranges (KDE 50%). For each cuckoo, the average percentage of overlap was calculated as well as the number of home ranges it overlapped with (in total, for more than 30% and for more than 50%).

### Statistical analysis

Regional and sex differences in home range size or NDVI were assessed using ANOVA tests and Tukey’ Honest Significant Differences for post-hoc comparisons (regional analyses only). A two-sample t-test of means was used to test for a difference in breeding range NDVI between cuckoos that left or stayed in the breeding area. Population level differences in parasitism rates and breeding success were tested using the Chi-squared test. Normality of data was assessed and when assumptions were not met data were log-transformed to adhere to the normal distribution. Since this study and the one by Williams *et al*.^13^ of home ranges in the common cuckoo used similar techniques, a t-test was used to compare home range sizes between both cuckoo species.

There was a strong significant correlation between mean NDVI of MCP and KDE calculated home ranges (*r* = 0.92, *df* = 52, *p* < 0.0001). This shows that using MCPs gives an approximation of mean home range NDVI even though it does not reflect the actual size of the home range. To detect if the itineracy in the wintering area was related to food availability, a linear regression was performed between duration of stay in each sub-Saharan home range and the average NDVI. MCP home ranges were used for this analysis to include those stop-overs where the cuckoos spent little time, since these could potentially reflect unsuitable home ranges. In addition, we performed a linear regression between KDE home range size and mean NDVI to assess whether food availability plays a role in determining home range size.

To see which parameter best explains home range size (KDE 95%), generalized linear mixed-effect models (GLMM) were made. Since home range size best fitted a lognormal distribution, the models were formed using a Gaussian distribution and a log-link function. Models included (one, all, or a combination of) the (untransformed) explanatory variables region (breeding, non-breeding Spain, wintering), sex and NDVI, as well as all interactions between them. Since multiple home ranges of the same birds were included, bird identity was included as a random factor. Additionally, since we found sample size to be strongly predictive of home range size, the number of fixes recorded per home range (sample size) and the mean quality of fixes per home range were added as nuisance factors in all models^13^. Variables were scaled where appropriate for better comparability. Models were compared using second-order Akaike information criterion (AICc), this metric was chosen as it is more conservative than AIC, penalizing low sample size and the use of additional parameters more strongly^64^. ANOVA Type II Wald chi-square tests were used to test for significance. The analyses were performed in R 3.2.4 using the packages ‘car^65^’, ‘rgeos^66^’, ‘lme4^67^’ and ‘MuMIn^68^’.

### Ethics statement

All research has been conducted according to relevant Spanish national (Real Decreto 1201/2005, de 10 de Octubre) and regional guidelines. All necessary permits (including ethical approval) were obtained from the Consejería de Medio Ambiente de la Junta de Andalucia, Spain (SGMN/GyB/JMIF).

## Supplementary information

Supplementary Information

Supplementary Information 2

## Data Availability

Data is available as supplementary material.
