# Supplementary material for: Territoriality and variation in home range size through the entire annual range of migratory great spotted cuckoos (Clamator glandarius)
Source: Sci Rep. 2019 Apr 17;9:6238. doi: 10.1038/s41598-019-41943-2 (PMC6470221; doi:10.1038/s41598-019-41943-2)

Supplementary information associated with the manuscript: Territoriality and variation in home range size through the entire annual range of migratory great spotted cuckoos (*Clamator glandarius*) by Josse Ruhmann, Manuel Soler, Tomás Pérez-Contreras, Juan Diego Ibáñez-Álamo.

**Table S1:** Steps undertaken in data preparation.

| *Steps taken* | *Number of Fixes* | *%* |
| --- | --- | --- |
| Total number of received Argos location fixes | 84.596 |  |
| *Removal of duplicate fixes* | -63.643 |  |
| *Removal of fixes with exact 1/2 h difference* | -2.949 |  |
| Number of unique fixes | 18.004 | 100.0% |
| *Removal of low quality fixes (0, A, B, Z)* | -11.067 | 61.5% |
| *Removal of spatial autocorrelation (<5 min difference)* | -529 | 2.9% |
| *Removal of fixes not part of a home range* | -508 | 2.8% |
| Number of fixes used in home range calculation | 5.900 | 32.8% |

**Table S2:** Home ranges used in the study.

* = first date of tracking, † = bird lost, ^ = exact date uncertain

| ***#*** | ***Cuckoo*** | ***Sex*** | ***Year*** | ***Dates*** | ***Duration (days)*** | ***Area*** | ***Nr of fixes*** | ***Mean Fix Quality*** | ***KDE 95% (km2)*** | ***KDE 50% (km2)*** | ***MCP 95% (km2)*** | ***NDVI (MCP)*** |
| --- | --- | --- | --- | --- | --- | --- | --- | --- | --- | --- | --- | --- |
| 1 | Black | M | 2014 | 9* - 20 Apr† | 11.3 | Breeding Area | 42 | 1.8 | 30.2 | 5.4 | 20.7 | 90.8 |
| 2 | Blue | F | 2014 | 29 Apr* - 4 Jul^ | 57.4 | Breeding Area | 157 | 1.6 | 65.1 | 9.7 | 88.4 | 83.5 |
| 3 | Blue | F | 2014 | 11 Jul - 25 Jul | 13.5 | Morocco | 42 | 1.5 | 99.4 | 20.6 | 122.9 | 75.1 |
| 4 | Brown | M | 2014 | 8 Apr -24 May* | 46.4 | Breeding Area | 244 | 1.7 | 33.5 | 4.0 | 98.6 | 90.5 |
| 5 | Brown | M | 2014 | 27 - 29 May | 2.7 | Central Spain | 15 | 1.6 | - | - | 19.2 | 108.0 |
| 6 | Brown | M | 2014 | 31 May - 10 Jul | 40.5 | Central Spain | 155 | 1.8 | 27.6 | 3.4 | 30.3 | 104.0 |
| 7 | Brown | M | 2014 | 11- 20 Jul | 9.1 | Central Spain | 34 | 1.9 | 25.1 | 4.3 | 11.6 | 98.3 |
| 8 | Brown | M | 2014 | 25 Jul - 7 Aug† | 13.0 | Morocco | 39 | 1.9 | 30.4 | 5.2 | 17.5 | 80.6 |
| 9 | Dark Blue | F | 2013 | 4* -29 May | 25.1 | Breeding Area | 59 | 2.0 | 18.0 | 2.7 | 28.5 | 104.5 |
| 10 | Dark Blue | F | 2013 | 2 - 9 Jun | 7.0 | Breeding Area | 23 | 1.9 | - | - | 1.9 | 106.5 |
| 11 | Dark Blue | F | 2013 | 16 Jun - 9 Jul | 22.9 | Central Spain | 64 | 1.9 | 12.1 | 2.2 | 5.6 | 116.4 |
| 12 | Dark Blue | F | 2013 | 16 - 31 Jul† | 15.8 | Morocco | 26 | 1.7 | - | - | 11.5 | 108.1 |
| 13 | Green | F | 2014 | 8 Apr* - 13 Jun | 65.6 | Breeding Area | 255 | 1.6 | 49.6 | 6.1 | 100.0 | 92.5 |
| 14 | Green | F | 2014 | 15 Jun - 29 Jun | 14.0 | Morocco | 52 | 1.5 | 51.4 | 7.8 | 37.4 | 86.3 |
| 15 | Green | F | 2014 | 5 - 8 Jul | 3.8 | Coastal Africa | 14 | 2.2 | - | - | 3.4 | 80.8 |
| 16 | Green | F | 2014 | 9 - 30 Jul^ | 17.7 | Coastal Africa | 44 | 1.8 | 11.9 | 2.2 | 19.3 | 90.2 |
| 17 | Green | F | 2014 | 31 Jul - 15 Aug | 15.7 | Coastal Africa | 60 | 1.8 | 32.6 | 5.7 | 22.0 | 89.4 |
| 18 | Green | F | 2014 | 19 Aug - 31 Aug | 12.1 | Inner Africa | 38 | 1.7 | 20.3 | 3.4 | 14.3 | 110.4 |
| 19 | Green | F | 2014 | 1 - 7 Sep | 5.7 | Inner Africa | 19 | 1.7 | - | - | 3.7 | 107.4 |
| 20 | Green | F | 2014 | 8 - 23 Sep^ | 10.4 | Inner Africa | 43 | 1.6 | 64.1 | 9.6 | 36.9 | 109.5 |
| 21 | Green | F | 2014 | 25 Sep - 16 Oct | 21.0 | Inner Africa | 54 | 2.0 | 25.2 | 4.2 | 16.2 | 92.6 |
| 22 | Green | F | 2014 | 18 Oct - 15 Feb^ | 118.7 | Inner Africa | 280 | 1.7 | 125.4 | 17.3 | 162.6 | 76.6 |
| 23 | Light Blue | M | 2013 | 13 May* - 26 Jun†^ | 19.9 | Breeding Area | 83 | 1.8 | 47.2 | 7.6 | 95.9 | 106.3 |
| 24 | Light Blue | M | 2013 | 3 - 14 Jun | 11.7 | Non-Breeding Spain | 26 | 1.5 | - | - | 18.4 | 103.4 |
| 25 | Light Green | F | 2013 | 13 May* - 5 Jun | 23.2 | Breeding Area | 80 | 2.0 | 30.5 | 4.4 | 40.2 | 110.9 |
| 26 | Light Green | F | 2013 | 9 - 19 Jun | 9.5 | Breeding Area | 35 | 2.1 | 15.8 | 3.9 | 6.9 | 108.8 |
| 27 | Light Green | F | 2013 | 28 Jun - 13 Jul† | 15.1 | Non-Breeding Spain | 37 | 1.8 | 12.0 | 2.0 | 8.3 | 104.6 |
| 28 | Lime | M | 2014 | 8 Apr* - 12 Jun^ | 56.2 | Breeding Area | 308 | 2.0 | 70.2 | 10.1 | 125.7 | 98.1 |
| 29 | Orange-2 | M | 2014 | 29 Apr* - 8 May | 9.4 | Breeding Area | 35 | 1.7 | 14.2 | 2.5 | 11.1 | 95.7 |
| 30 | Orange-2 | M | 2014 | 11 May - 27 May | 16.6 | Non-Breeding Spain | 44 | 1.8 | 14.0 | 2.8 | 8.7 | 128.7 |
| 31 | Orange-2 | M | 2014 | 30 May - 24 Jun | 25.4 | Central Spain | 46 | 1.8 | 13.9 | 2.5 | 9.5 | 108.1 |
| 32 | Orange-1 | M | 2013 | 14 May* - 22 Jun | 39.5 | Breeding Area | 106 | 1.7 | 24.9 | 3.8 | 55.1 | 102.9 |
| 33 | Orange-1 | M | 2013 | 25 Jun - 2 Jul† | 7.1 | Non-Breeding Spain | 18 | 1.5 | - | - | 17.6 | 84.5 |
| 34 | Pink | F | 2014 | 8 Apr* - 25 May | 46.3 | Breeding Area | 268 | 1.8 | 41.2 | 6.3 | 66.8 | 94.5 |
| 35 | Pink | F | 2014 | 25 May - 26 Jun | 30.5 | Non-Breeding Spain | 79 | 1.6 | 23.0 | 3.3 | 23.1 | 129.4 |
| 36 | Purple | M | 2014 | 29 Apr* - 8 Jun | 40.2 | Breeding Area | 200 | 1.6 | 47.0 | 5.3 | 219.5 | 87.1 |
| 37 | Purple | M | 2014 | 9 Jun - 1 Jul | 22.2 | Breeding Area | 111 | 1.6 | 59.2 | 7.9 | 56.7 | 84.3 |
| 38 | Purple | M | 2014 | 7 - 19 Jul | 12.1 | Morocco | 37 | 1.6 | 15.6 | 2.8 | 10.0 | 77.1 |
| 39 | Purple | M | 2014 | 23 Jul - 8 Aug | 16.3 | Coastal Africa | 68 | 1.9 | 13.1 | 2.0 | 16.3 | 100.0 |
| 40 | Purple | M | 2014 | 15 Aug - 25 Aug | 10.2 | Inner Africa | 41 | 2.0 | 11.8 | 1.6 | 26.9 | 154.5 |
| 41 | Purple | M | 2014 | 28 Aug - 9 Sep† | 11.2 | Inner Africa | 50 | 1.7 | 27.6 | 4.4 | 23.1 | 97.3 |
| 42 | Red | M | 2013 | 2* - 7 May | 4.7 | Breeding Area | 24 | 1.8 | - | - | 8.0 | 113.9 |
| 43 | Red | M | 2013 | 13 May - 4 Jun^ | 53.1 | Breeding Area | 56 | 1.9 | 42.5 | 9.1 | 60.5 | 122.9 |
| 44 | Red | M | 2013 | 7 Jun - 21 Jul | 44.1 | Central Spain | 135 | 2.0 | 16.7 | 1.7 | 45.4 | 115.6 |
| 45 | Red | M | 2013 | 27 - 29 Jul | 2.3 | Coastal Africa | 8 | 1.5 | - | - | 3.3 | 70.8 |
| 46 | Red | M | 2013 | 7 Aug - 24 Sep | 47.5 | Coastal Africa | 140 | 2.0 | 11.9 | 2.3 | 28.7 | 143.7 |
| 47 | Red | M | 2013 | 30 Sep - 9 Oct | 9.1 | Inner Africa | 18 | 1.9 | - | - | 1.2 | 91.0 |
| 48 | Red | M | 2013 | 11 - 21 Oct | 9.2 | Inner Africa | 20 | 2.2 | - | - | 4.7 | 100.6 |
| 49 | Red | M | 2013 | 23 Oct - 12 Nov | 20.8 | Inner Africa | 45 | 2.1 | 16.3 | 2.2 | 13.5 | 86.3 |
| 50 | Red | M | 2013 | 15 - 19 Nov | 4.7 | Inner Africa | 15 | 2.3 | - | - | 3.9 | 86.0 |
| 51 | Red | M | 2013 | 22 Nov - 18 Feb | 88.8 | Inner Africa | 97 | 2.2 | 7.2 | 0.9 | 6.3 | 80.6 |
| 52 | Red | M | 2014 | 1 - 6 Mar | 4.4 | Morocco | 14 | 2.1 | - | - | 2.2 | 121.2 |
| 53 | Red | M | 2014 | 6- 15 Mar | 8.9 | Morocco | 22 | 2.0 | - | - | 2.9 | 128.3 |
| 54 | Red | M | 2014 | 19 Mar - 3 Jun | 75.6 | Breeding Area | 244 | 1.8 | 31.7 | 3.9 | 61.1 | 87.0 |
| 55 | Red | M | 2014 | 9 Jun - 6 Jul | 27.0 | Central Spain | 64 | 1.8 | 11.3 | 1.3 | 21.5 | 101.3 |
| 56 | Red | M | 2014 | 17 - 24 Jul | 7.0 | Coastal Africa | 22 | 2.0 | - | - | 1.5 | 75.0 |
| 57 | Red | M | 2014 | 4 - 6 Aug | 2.3 | Inner Africa | 9 | 1.8 | - | - | 7.4 | 73.3 |
| 58 | Red | M | 2014 | 9 - 16 Aug | 6.9 | Inner Africa | 16 | 1.9 | - | - | 6.7 | 88.2 |
| 59 | Red | M | 2014 | 27 Aug - 9 Sep | 13.4 | Inner Africa | 28 | 1.8 | - | - | 10.8 | 108.7 |
| 60 | Red | M | 2014 | 11 - 1 Oct^ | 17.7 | Inner Africa | 34 | 2.1 | 13.2 | 2.7 | 11.7 | 92.9 |
| 61 | Red | M | 2014 | 6 - 8 Oct | 2.5 | Inner Africa | 10 | 2.4 | - | - | 1.8 | 87.3 |
| 62 | Red | M | 2014 | 10 - 12 Oct | 2.2 | Inner Africa | 4 | 1.5 | - | - | - | - |
| 63 | Red | M | 2014 | 25 Oct - 3 Nov | 9.3 | Inner Africa | 23 | 2.0 | - | - | 6.4 | 91.2 |
| 64 | Red | M | 2014 | 7 Nov - 20 Dec | 43.1 | Inner Africa | 83 | 1.9 | 44.4 | 9.1 | 35.2 | 79.7 |
| 65 | Red | M | 2014 | 25 Dec - 10 Feb | 46.8 | Morocco | 107 | 2.0 | 14.8 | 2.8 | 18.1 | 157.1 |
| 66 | Red | M | 2015 | 14 Feb - 28 Apr | 73.0 | Breeding Area | 172 | 1.8 | 23.5 | 3.1 | 27.9 | 86.5 |
| 67 | Red | M | 2015 | 4 May - 13 Jun | 39.6 | Central Spain | 91 | 1.8 | 40.4 | 5.3 | 38.0 | 117.7 |
| 68 | Red | M | 2015 | 15 Jun - 12 Jul | 26.8 | Central Spain | 47 | 1.9 | 9.9 | 2.0 | 7.0 | 98.0 |
| 69 | Red | M | 2015 | 23 Jul - 1 Aug | 9.2 | Coastal Africa | 32 | 1.8 | 11.7 | 2.2 | 9.3 | 92.7 |
| 70 | Red | M | 2015 | 6 - 8 Aug | 2.6 | Coastal Africa | 13 | 2.4 | - | - | 12.2 | 77.3 |
| 71 | Red | M | 2015 | 10 - 13 Aug | 2.5 | Coastal Africa | 10 | 1.9 | - | - | 9.7 | 80.6 |
| 72 | Red | M | 2015 | 17 Aug - 15 Sep | 29.4 | Coastal Africa | 76 | 2.3 | 4.0 | 0.7 | 6.1 | 112.5 |
| 73 | Red | M | 2015 | 17 Sep - 10 Oct | 22.6 | Coastal Africa | 46 | 2.2 | 13.9 | 2.2 | 11.3 | 95.6 |
| 74 | Red | M | 2015 | 16 Oct - 3 Nov | 18.1 | Inner Africa | 17 | 2.1 | - | - | 2.4 | 87.4 |
| 75 | Red | M | 2015 | 8 Nov - 19 Jan^ | 56.8 | Inner Africa | 56 | 2.3 | 4.8 | 0.8 | 3.6 | 79.9 |
| 76 | Red | M | 2015 | 24 Nov - 5 Dec | 11.5 | Inner Africa | 13 | 1.9 | - | - | 1.5 | 79.2 |
| 77 | Red | M | 2016 | 24 - 26 Jan | 2.3 | Morocco | 3 | 2.3 | - | - | - | - |
| 78 | Red | M | 2016 | 28 - 31 Jan | 2.4 | Morocco | 3 | 2.0 | - | - | - | - |
| 79 | Red | M | 2016 | 2 - 20 Feb | 18.2 | Morocco | 7 | 2.3 | - | - | 0.2 | 67.7 |
| 80 | Red | M | 2016 | 27 Feb -29 Apr | 61.8 | Breeding Area | 162 | 1.8 | 17.9 | 2.5 | 30.0 | 76.3 |
| 81 | White | F | 2014 | 9 Apr* - 29 Jun† | 81.5 | Breeding Area | 314 | 1.6 | 68.5 | 10.3 | 75.3 | 87.9 |
| 82 | Yellow | M | 2014 | 9 Apr* - 24 May† | 23.3 | Breeding Area | 169 | 1.8 | 139.8 | 26.1 | 163.9 | 87.0 |

**Figure S1:** Overview of different calculation techniques for two home ranges in the breeding area. (A) Raw data fixes as received from Argos. Yellow lines are error ellipses. Home ranges calculated using 95% Minimum convex polygons (B), 95% Kernel Density Estimation (C) and 50% KDE (D). The satellite imagery was obtained from Google Earth 7.1 (Google Inc., CA, USA).


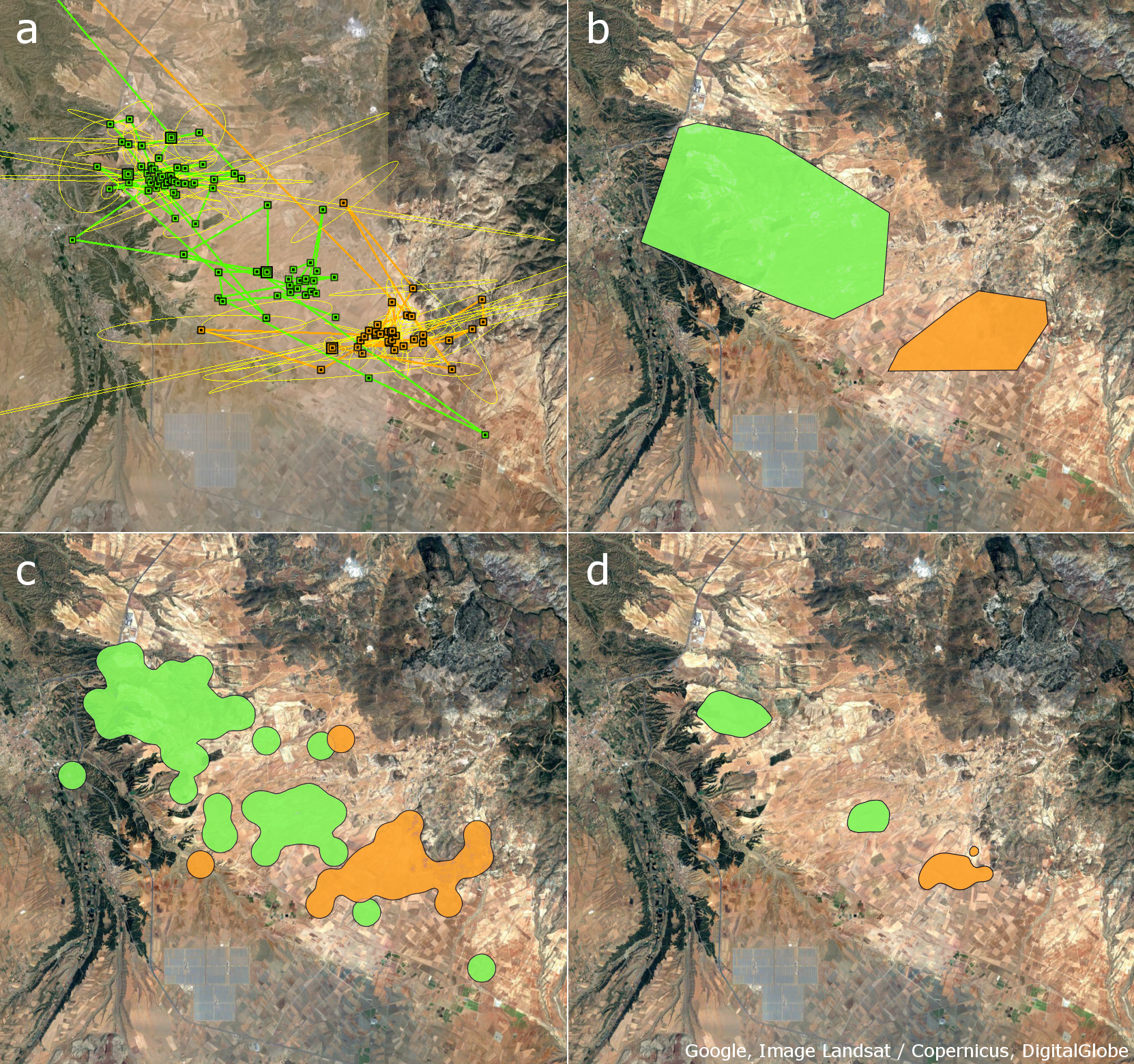

Supplement: Supplementary file 1 — Supplementary Information [file 41598_2019_41943_MOESM1_ESM.docx]
